# Green Extract from Pre-Harvest Tobacco Waste as a Non-Conventional Source of Anti-Aging Ingredients for Cosmetic Applications

**DOI:** 10.3390/plants14142189

**Published:** 2025-07-15

**Authors:** Mariana Leal, María A. Moreno, María E. Orqueda, Mario Simirgiotis, María I. Isla, Iris C. Zampini

**Affiliations:** 1 Instituto de Bioprospección y Fisiología Vegetal (INBIOFIV-CONICET-UNT), San Martín 1545, San Miguel de Tucumán T4000, Tucumán, Argentina; maariileal@hotmail.com (M.L.); .; eorqueda@yahoo.com.ar (M.E.O.); misla@csnat.unt.edu.ar (M.I.I.); 2 Facultad de Ciencias Naturales e IML, Universidad Nacional de Tucumán (UNT), Miguel Lillo 205, San Miguel de Tucumán T4000, Tucumán, Argentina; 3 Instituto de Farmacia, Facultad de Ciencias, Universidad Austral de Chile, Campus Isla Teja, Valdivia 5090000, Chile; mario.simirgiotis@uach.cl

**Keywords:** *Nicotiana tabacum* L., pre-harvest waste, NaDES, anti-inflammatory, anti-aging, cosmetics

## Abstract

The cigarette production from *Nicotiana tabacum* generates significant amounts of waste, with an estimated 68.31 million tons of pre- and post-harvest waste discarded annually. The pre-harvest waste includes the upper parts of the plant, inflorescences, and bracts, which are removed to help the growth of the lower leaves. This study explores the potential of apical leaves from *Nicotiana tabacum* var. Virginia, discarded during the budding stage (pre-harvest waste). The leaves were extracted using environmentally friendly solvents (green solvents), including distilled water (DW) and two natural deep eutectic solvents (NaDESs), one consisting of simple sugars, fructose, glucose, and sucrose (FGS) and the other consisting of choline chloride and urea (CU). The anti-inflammatory and anti-aging potential of these green extracts was assessed by the inhibition of key enzymes related to skin aging. The xanthine oxidase and lipoxygenase activities were mostly inhibited by CU extracts with IC_50_ values of 63.50 and 8.0 μg GAE/mL, respectively. The FGS extract exhibited the greatest hyaluronidase inhibition (49.20%), followed by the CU extract (33.20%) and the DW extract (20.80%). Regarding elastase and collagenase inhibition, the CU extract exhibited the highest elastase inhibition, while all extracts inhibited collagenase activity, with values exceeding 65%. Each extract had a distinct chemical profile determined by LC-ESI-QTOF-MS/MS and spectrophotometric methods, with several shared compounds present in different proportions. CU extract is characterized by high concentrations of rutin, nicotiflorin, and azelaic acid, while FGS and DW extracts share major compounds such as quinic acid, fructosyl pyroglutamate, malic acid, and gluconic acid. Ames test and *Caenorhabditis elegans* assay demonstrated that at the concentrations at which the green tobacco extracts exhibit biological activities, they did not show toxicity. The results support the potential of *N. tabacum* extracts obtained with NaDESs as antiaging and suggest their promising applications in the cosmetic and cosmeceutical industries.

## 1. Introduction

Tobacco *(Nicotiana tabacum* L.) belongs to the family *Solanaceae*, and it is a resource of significant economic importance for many countries; with seven of the world’s largest producers located on the American continent. Between 1980 and 2012, global cigarette consumption increased by 26%, indicating that the tobacco market was still expanding [1].

There are several tobacco varieties based on their curing process, namely, sun-cured, flue-cured, air-cured, and fire-cured. In Argentina, between 63,000 and 75,000 hectares are cultivated with approximately ten types of tobacco. Virginia (flue-cured) and Burley (air-cured) are the most important tobacco varieties, accounting for over 90% of the national production. Virginia tobacco represents about 60% of the total production and is primarily grown in the northwest, specifically in the provinces of Salta and Jujuy. Burley tobacco accounts for approximately 35% of production and is predominantly cultivated in Misiones and Tucumán. The “criollo” types are characteristic of the northeast of the country [2]. Good agricultural practices are used in Virginia tobacco production, in accordance with the standards of the Cooperation Centre for Scientific Research Relative to Tobacco (CORESTA) [3]. These practices guarantee crop quality and meet international market requirements. It is not until tobacco plants reach the end of their growth phase that they begin to bloom. Flowers are necessary on tobacco plants if the intention is to produce tobacco seeds. However, blooming is undesirable as it results in thinner leaves, which can reduce yield and leaf quality. To overcome this problem, tobacco growers perform a process called ‘topping’ or ‘trimming’. This involves removing the flowers and apical leaves before the harvest begins [4]. The apical leaves and flowers are left in the field as waste, constituting pre-harvest waste.

The plant density of Virginia tobacco is around 20,000 plants per hectare, generating approximately 2000 Kg.ha^−1^ of pre-harvest biomass waste [2]. Therefore, the production of Virginia tobacco in Argentina generates an estimated 140,000 tons of pre-harvest waste.

The concept of a circular economy is becoming increasingly relevant, as producing large amounts of waste without finding ways to reuse it is no longer sustainable. In this context, tobacco pre-harvest waste, consisting of unused biomass from cigarette tobacco production, could be repurposed for use in cosmetics or pharmaceuticals. Indeed, tobacco inflorescence and leaf extracts are referenced in the Cosmetic Ingredient Database, CosIng [5], as cosmetic ingredients.

Traditionally, organic solvents have been used to obtain various specialized compounds from tobacco leaves such as phenolic compounds [6,7,8,9,10], sterols [11], terpenes [12], and alkaloids [6,13] that would contribute to its potential use. However, these solvents are relatively toxic, potentially leaving harmful waste in the products extracted, thus contributing to air pollution and global warming [14].

The drawbacks of conventional organic solvents can be addressed by using green solvents or non-conventional solvents, such as natural deep eutectic solvents (NaDESs), which are eutectic mixtures composed of natural substances with a particular molar ratio, such as sugars, organic acids and bases, and amino acids. Their supramolecular structure allows them to melt at a unique, well-defined temperature known as the “eutectic point,” or the lowest melting point for a given composition. These solvents offer several advantages over conventional solvents, including low cost, biodegradability, ease of production, renewability, absence of toxicity, and high stability [15]. They are also considered environmentally friendly [16]. The preparation of NaDESs yields easy results with high purity and no waste formation [17] according to the fundamental principles of green chemistry [16].

These characteristics pave the way for the development of innovative “green extracts” with distinct phytochemical profiles and biological activities, suitable for use in cosmetic, medicinal, food, and textile applications [15,16,18].

In previous works, conventional solvents, such as water, acetone, ethanol, and non-conventional solvents like NaDESs—i.e., sucrose:lactic acid (LAS), fructose:glucose:sucrose (FGS), lactic acid:sucrose:water (SALA), choline chloride:urea (CU), and citric acid: propylene glycol (CAP)—were used for extraction of some antioxidants from tobacco pre-harvest waste that could be used in green cosmetic formulation to protect the skin from oxidation [19,20].

The main causes of skin aging are oxidative stress and enhanced activation of proteolytic enzymes [21]. Matrix metalloproteinases, such as collagenase and elastase, degrade the collagen network and elastin fibers, respectively, damaging the extracellular matrix [22]. Hyaluronic acid, which is essential for water retention and maintaining the smooth, hydrated, lubricated, and moisturized skin tissues, is broken down by the enzyme hyaluronidase. Therefore, the hydrolysis of hyaluronic acid causes skin to dry out, resulting in a loss of smoothness and elasticity [23]. Furthermore, the inflammatory process is characterized by increased oxidative stress and the overproduction of ROS, which is mediated by various pro-inflammatory enzymes. These include cyclooxygenases, which produce prostaglandins and thromboxane, and lipoxygenase (LOX), that produces leukotrienes and lipoxins [24]. Furthermore, xanthine oxidase (XOD), is an enzyme that is involved in the metabolism of xanthine and hypoxanthine, and it catalyzes the oxidation of these compounds to produce superoxide anion, which is a highly reactive oxygen species. These processes contribute to wrinkling, decreased skin flexibility and, ultimately, skin aging [22].

In the field of cosmetic care, the compounds with potential capacity to interfere in the evolution of aging that involve inflammatory processes, are of great interest. Moreover, inhibitors of collagenase, elastase, and hyaluronidase are key in cosmetic formulations that are designed to reduce or delay signs of skin aging.

Although the extraction of specialized metabolite groups from tobacco leaves using NaDESs has been described [19], to date, they have not yet been identified and their potential use in anti-aging cosmetics has not yet been demonstrated.

Based on these considerations, the present study aims to evaluate the activity on pro-inflammatory enzymes, the activity on skin aging-related enzymes, and the chemical composition and toxicity of standardized extracts obtained from leaf waste of *Nicotiana tabacum* var. Virginia by using green solvents (NaDESs).

## 2. Results and Discussion

**Figure 1:**
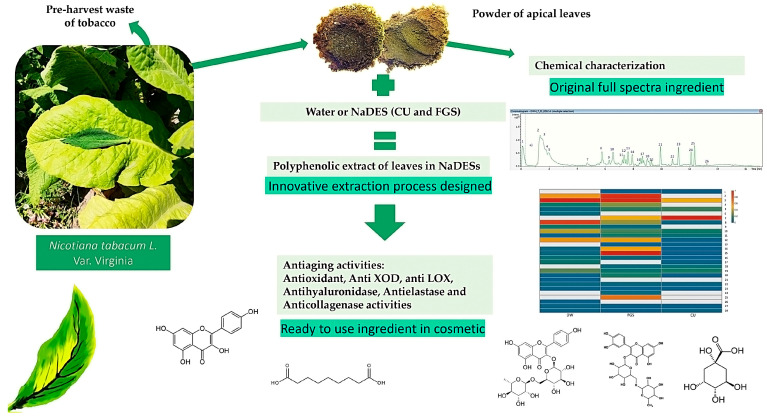
Flowchart showing an innovative method for extracting cosmetic ingredients from *Nicotiana tabacum* apical leaves using non-conventional solvents (NaDESs)—chemical and functional characterization of cosmetic ingredients.

In a previous paper, the potential of five NaDESs—i.e., sucrose:lactic acid (LAS), fructose:glucose:sucrose (FGS), lactic acid:sucrose:water (SALA), choline chloride:urea (CU), and citric acid:propylene glycol (CAP)—as solvents for the extraction of metabolites from apical leaves of *Nicotiana tabacum* var. Virginia was determined; these leaves are discarded during the budding of the plants (pre-harvest) [19]. The potency was compared with that of traditional solvents such as acetone:water; 70% ethanol, and water. FGS and CU were the more effective NaDESs for phenolic compounds and total flavonoid extraction from tobacco leaves. So, given the significance of adopting green chemistry principles in the cosmetic sector, we recognized the need to develop an effective, eco-friendly extraction method enriched with phytochemicals with potential anti-aging effects on the skin. For this, CU and FGS were selected in the present study to extract bioactive specialized metabolites from apical leaves of *Nicotiana tabacum*, discarded during the topping of the tobacco plants (pre-harvest waste). The extracts obtained with NaDESs were then compared with the aqueous extract and subjected to chemical and functional characterization (Figure 1).

### 2.1. Phytochemical Composition of Nicotiana tabacum Green Extracts

The total phenolic compounds and total flavonoid content were determined in extracts freshly prepared using distilled water, CU, and FGS as solvents (Table 1). The TPC and flavonoid contents were higher in the extract obtained with CU, followed by FGS, whereas the extraction yield with water was low.

**Figure 2:**
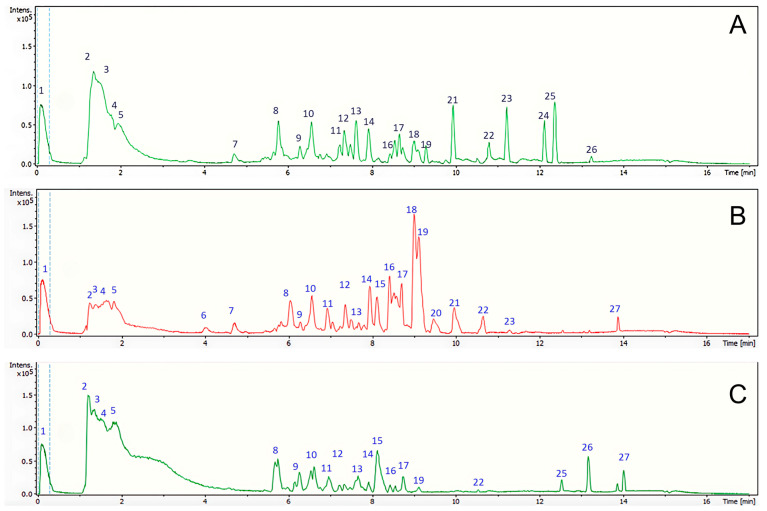
UHPLC/ESI/MS/MS chromatograms: (**A**) *Nicotiana tabacum* aqueous extract (DW-E), (**B**) *N. tabacum* CU extract (CU-E), (**C**) *N. tabacum* FGS extract (FGS-E).

The green extracts were analyzed by LC-ESI-QTOF-MS/MS and 27 chemical compounds were tentatively identified. The chromatograms of the chemical profile of the extracts are shown in Figure 2. Each extract shows a characteristic chemical profile, with several compounds in common that are observed in different proportions in each sample. The aqueous and FGS extracts were enriched into more polar compounds, eluting in the first few minutes, while the CU extract was enriched in compounds of intermediate polarity (eluting between 8 and 10 min).

The identified compounds, including 19 in the CU extract, 23 in the FGS extract, and 21 in the aqueous extract of *N. tabacum* apical leaves waste. The metabolites identified were mainly organic acids, namely phenolic acid, fatty acids, and amino acids, as well as several flavonoids and one alkaloid. In the conditions in which the analysis of the chemical composition of tobacco leaves extracts was carried out, no chemical structures corresponding to pesticides were detected, probably because the pre-harvest tobacco waste included in the present study comes from tobacco crops grown for export, i.e., cultivated using the good agricultural practices recommended by CORESTA. Table 2 shows a detailed list of the identified compounds. Several of these metabolites, such as rutin, nicotiflorin, quinic acid, malic acid, citric acid, 2-isopropylmalic acid, aspartate, phenylacetic acid, feruloyltyramine, abscisic acid, sebacic acid, quercetin, and kaempferol, were previously reported as tobacco leaf extracts obtained with conventional solvents [6,8,25,26,27,28,29,30,31,32,33,34,35]. However, this is the first report of these compounds extracted using green solvents, such as NaDESs. Additionally, the study also identified N-fructosyl pyroglutamate, phenylacetyl aspartic acid, citramalic acid, theophylline, and tricin, which had not been previously described in *N. tabacum* extracts.

**Figure 3:**
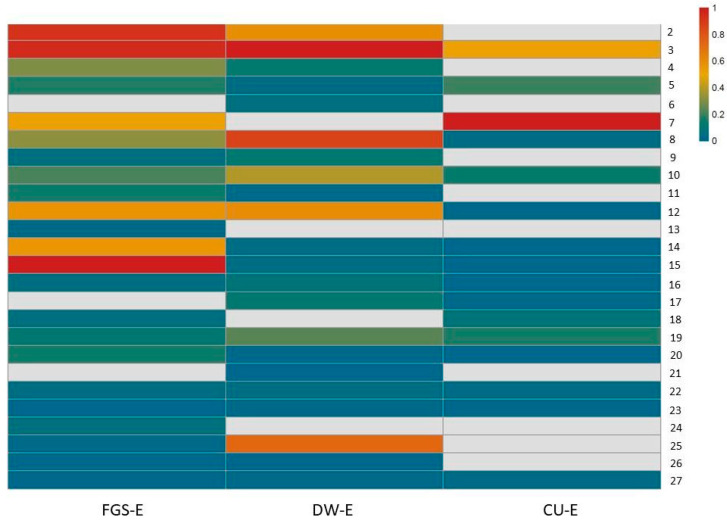
Heatmap represents the relative abundance of each compound in the different extracts from *Nicotiana tabacum* apical leaves obtained with DW: distilled water; NaDES: FGS and CU; fructose:glucose:sucrose (FGS), choline chloride:urea (CU). DW-E: extract in water; FGS-E (extract in FGS) and CU-E (extract in CU). The numbers from 2 to 27 on the right indicate each of the compounds identified in the extracts according to Table 2. The amount of each compound is represented by the color intensity from 0 to 1.

Due to the exploratory nature of the study, no absolute quantification was performed. A heatmap (Figure 3) was devised, where the rows represent compounds identified by UHPLC/ESI/MS/MS and the matrix columns represent the extracts obtained with different solvents. The results are visualized with a color scale, with red indicating an increase and blue indicating a decrease in compound values across the various extracts. The values used correspond to the signal intensity values of each compound provided by metabolomic analysis, values which are directly proportional to the analyte quantity detected. The blank cells correspond to the absence of a signal for that compound. Subsequently, these values were normalized to construct the heatmap. As shown in the heatmap, CU extract is characterized by high concentrations of rutin (peak 7), malic acid (peak 3), citric acid (peak 5), and azelaic acid (peak 19). DW and FGS extracts share major compounds such as quinic acid (peak 2) and malic acid (peak 3). Additionally, DW contains high levels of phenylacetyl aspartic acid (peak 10), while FGS is enriched in aspartate (peak 14), succinic acid (peak 15), and kaempferol (peak 25).

Several of these compounds identified are relevant for use in the cosmetic industry, since they exhibit biological activities, such as anti-inflammatory, antioxidant and anti-aging properties. For example, rutin, one of the most abundant flavonoids in tobacco leaves, has demonstrated antioxidant [36], anti-elastase, anti-tyrosinase, and anti-hyaluronidase [37] activities. Nicotiflorin has been reported to possess antioxidant, anti-inflammatory [38], and anti-hyaluronidase activities [39]. Azelaic acid is widely used in cosmetics [40] and has demonstrated anti-inflammatory [41], antiacne [42], and antibacterial [43] activities. Quinic acid has been recognized for its antioxidant, antimicrobial [44], anti-inflammatory [45], and anti-tyrosinase properties [46]. Malic acid [47] and citric acid [48] also exhibit antioxidant properties. Gluconic acid is recognized for its detoxifying, antioxidant, and anti-inflammatory effects. It is also a precursor of vitamin C, a well-known antioxidant agent. As an organic acid widely used in cosmetic and food industries, gluconic acid exhibits strong chelating properties and possesses antiseptic activity [49]. Kaempferol has been reported to exhibit antimicrobial [50], anti-tyrosinase, and antioxidant properties [51]. Other compounds identified also show promising potential for cosmetic applications.

For instance, quercetin exhibits antioxidant, anticancer, antimicrobial, antidiabetic, and anti-inflammatory activities [52,53], as well as anti-tyrosinase effects [54]. Tricin has shown antioxidant [55], anticancer [56], and anti-aging activities [57]. Caffeoyl quinic acid has shown anti-inflammatory [58] and antioxidant activities [59]. Phenylacetic acid has been reported to possess antioxidant and anti-tyrosinase properties [60] and 10-hydroxystearic acid has demonstrated beneficial cosmetic properties by improving skin texture, reducing hyperpigmentation, and enhancing collagen synthesis through activation of peroxisome proliferator-activated receptor alpha (PPARα). This supports its use in anti-aging formulations [61]. Additionally, feruloyl tyramine exhibits antioxidant [62], antimicrobial, anti-melanogenesis, and anticancer activities [63]. Lastly, 2-isopropylmalic acid has demonstrated antimicrobial and antioxidant properties [64]. Because of this background, based on the chemistry of green tobacco extracts, it is worth evaluating their potential as anti-aging agents for use in cosmetics. It is also important to note that the extracts obtained are nicotine-free, and none of the identified specialized or primary metabolites are included in the lists in Annex II (banned cosmetic ingredients) or Annex III (restricted cosmetic ingredients) of Regulation No 1223/2009 (the Cosmetics Regulation) [65].

### 2.2. Effect of Tobacco Leaf Waste Extracts on Enzymatic Activities Related to Anti-Inflammatory Mechanisms

LOX and XOD are enzymes associated with both acute and chronic inflammatory conditions. Since inflammation induces oxidative stress and other effects that can have an impact on the proper system functioning over time, this chronic inflammation is thought to promote aging, called inflamm-aging [66]. This process contributes to the development of numerous diseases [67], including skin aging [21]. XOD catalyzes the oxidation of xanthine and hypoxanthine to uric acid and superoxide anion. By inhibiting XOD activity, ROS production is decreased, lowering the risk of oxidative stress and chronic inflammation. On the other hand, leukotrienes—molecules linked to acute inflammatory processes—are produced by mediators of the arachidonic acid cascade, including a pro-inflammatory enzyme such as LOX. Furthermore, it has been demonstrated that oxidative stress, tissue damage, and cell death can be caused by the dysregulation of certain LOX family enzymes [68].

This study demonstrates that the green extracts of *N. tabacum* apical leaf waste effectively inhibits both XOD and LOX activities. The IC_50_ values for the inhibition of these pro-inflammatory enzymes by aqueous and NaDESs extracts are presented in Table 3. Both enzymes were mostly inhibited by tobacco extract obtained with CU. The IC_50_ values for CU and FGS extract against XOD were 63.50 to 85.50 μg GAE/mL, respectively. The DW extract, at the maximum allowable concentration by the enzymatic method, exhibited no detectable activity. Allopurinol, a specific inhibitor of XOD, showed an IC_50_ of 50 μg/mL (Table 3). Regarding the inhibition of LOX enzyme, the tobacco leaf extract obtained with CU showed the highest activity (IC_50_ = 8.0 μg GAE/mL), whereas the DW and FGS extracts showed similar inhibitory effects (IC_50_ = 15.20 and 17.50 μg GAE/mL, respectively). The reference drug assayed, naproxen, inhibited LOX with an IC_50_ value of 14 μg/mL. Additionally, at the tested concentration, the NaDESs solvents alone did not inhibit the activity of XOD and LOX. Based on the results obtained, *N. tabacum* apical leaves may serve as a potential agent for preventing inflamm-aging. The inhibition of enzymatic activities related to anti-inflammatory mechanisms by *N. tabacum* leaf extracts may be attributed to the presence of bioactive compounds with demonstrated anti-inflammatory activity, such as quinic acid [69], caffeoylquinic acid [58,59], taxifolin [70], nicotiflorin [38], rutin [71], azelaic acid [41], quercetin [52], and kaempferol [72].

### 2.3. Activity on Skin Aging-Related Enzymes

Skin aging is characterized by the degradation of fibrous components of the dermal extracellular matrix, including elastin and collagen, as well as the loss of the oligosaccharides and proteoglycans, particularly hyaluronic acid and chondroitin sulphate glycosaminoglycan, which in turn impacts on the ability of skin to retain bound water [73]. Elastase and collagenase hydrolyze elastin and collagen, respectively, and an increased activity of these enzymes results in a decrease in skin resistance and the appearance of wrinkles. In addition, the hyaluronidase degrades hyaluronic acid into tiny fragments, which is linked to a decrease in skin hydration and the subsequent acceleration of skin aging [74].

**Figure 4:**
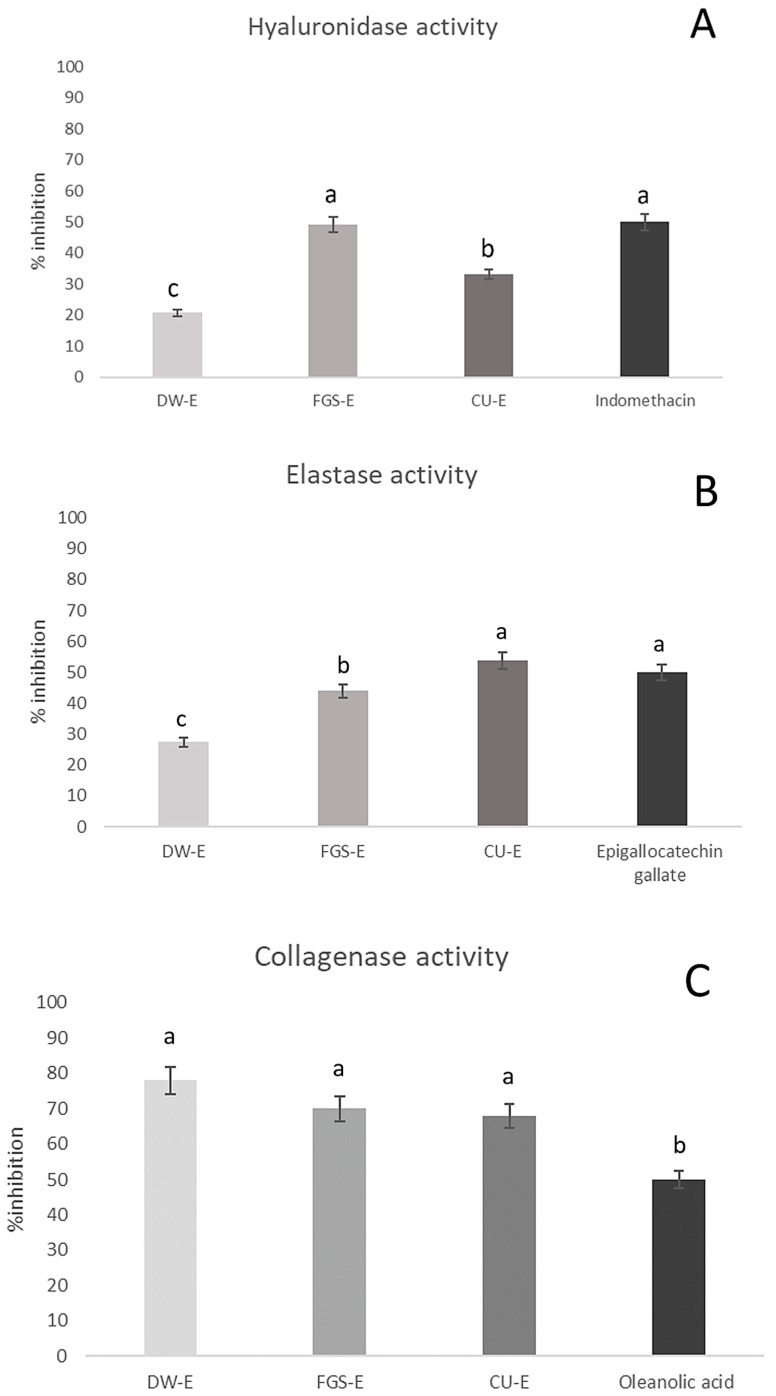
Effect of apical leaf waste of *Nicotiana tabacum* green extracts on hyaluronidase, elastase, and collagenase activity. DW-E: aqueous extract; FGS-E: extract in FGS solvent, and CU-E: extract in CU solvent; FGS: fructose:glucose:sucrose; CU: choline chloride:urea. (**A**) anti-hyaluronidase activity, (**B**) anti-elastase activity, and (**C**) anti-collagenase activity. In all cases, the maximum % inhibition of enzyme activity for each extract is included. The reference compounds are included in the graphs. Values are expressed as means ± standard deviation from triplicate measurements. Different letters indicate statistically significant differences among all samples, according to Tukey’s multiple comparison tests (*p* < 0.05).

In this study, the anti-aging potential of green extracts obtained from apical leaf waste of *N. tabacum* was evaluated through their inhibitory effects on key enzymes associated with skin aging. The inhibition percentages of skin aging-related enzyme activity by green extracts of tobacco leaves are shown in Figure 4. The FGS extract exhibited the highest inhibitory activity against hyaluronidase (50.00%), followed by the CU extract (33.20%) and the DW extract (20.80%). Indomethacin, used as a reference anti-aging compound, showed a similar inhibition percentage (50%) at 502 µg/mL. Notably, this is the first report of hyaluronidase inhibition by *N. tabacum* extracts obtained with NaDESs. Prommaba et al. (2022) [75] reported the anti-hyaluronidase activity only in tobacco leaf ethanolic extract, obtaining inhibition values like those achieved in this study for apical leaves of tobacco extracts using green solvent.

Regarding elastase and collagenase inhibition, the CU extract exhibited the highest elastase inhibition, reaching 53.8%, while all extracts (CU-E, FGS-E, and DW-E) inhibited collagenase activity, with values exceeding 65%. No significant differences were observed among the different extracts in terms of collagenase inhibition. Epigallocatechin gallate, used as a reference anti-elastase compound, showed 50% inhibition percentage at 50 µg/mL. Oleanolic acid, used as positive control of anti-collagenase activity, showed 50% inhibition percentage at 81.9 µg/mL. The high inhibitory capacity of NaDES extracts on collagenase could be ascribed to polyphenols with demonstrated anti-collagenase activity such as quercetin, quinic acid, fructosyl pyroglutamate compounds identified in FGS extract [54,76], and nicotiflorin, present in both NaDES extracts (FGS and CU) [77].

Other bioactive compounds identified in the *N. tabacum* extracts have also been associated with anti-aging effects (anti-elastase and anti-hyaluronidase), among them, caffeoylquinic acid (DW extract) and rutin (CU and FGS extracts) [37,58,76,78,79]. The presence of these active compounds demonstrates the anti-aging potential of *N. tabacum* extracts obtained with green solvents and suggests their promising applications in the cosmetic industry. Additionally, at the concentration tested, the NaDES solvents alone did not inhibit the activity of collagenase, elastase and hyaluronidase enzymes.

### 2.4. Toxicity Assays

Demonstrating the biological properties and identifying the chemical components of green extracts of tobacco is not sufficient to use them as anti-aging cosmetic ingredients. It is also necessary to comply with safety requirements related to innocuousness. This can be supported by toxicity studies. In the present study, two models were used: one based on prokaryotic organisms (*Salmonella* Typhymurium assay) and one based on eukaryotic organisms (*Caenorhabditis elegans* assay).

The Ames test can predict carcinogenicity with a high degree of accuracy (around 80%) employing various mechanisms, including frameshift mutations (TA98), base-pair substitutions (TA100), and point mutations. To ensure the safety of the extracts, their potential mutagenic effects were assessed. Since the mutagenicity ratio (MR) values were all below 2, none of the extracts caused any harm to the genetic material of the strains at the highest concentration tested (500 µg GAE/plate) (Table 4). Additionally, the NaDES solvents alone did not exhibit toxicity.

*C. elegans* is an excellent model organism used for aging research. This is due to its transparent body, which allows for anatomical observation, its high genetic homology (60.80%) with humans, the availability of its complete genome sequence, its conserved biological molecular responses, its high fertility rates (∼250 eggs/worm within several days), and its accessibility to molecular biology tools—i.e., transgenic techniques, gene knockouts, and RNAi knockdowns [80]. Furthermore, both the modest size of this organism and its three-week lifespan make it a good candidate for high throughput screening trials, reducing the cost of searching for anti-aging medications [81]. Moreover, there are no ethical issues with experiments involving *C. elegans*. These benefits have enabled *C. elegans* to contribute to numerous ground-breaking findings in the field of aging research [82]. Results are reported as a percentage of survival (Table 4). The tobacco leaves extracts were evaluated at increasing concentrations; the highest concentration tested was 100 µg GAE/mL. A survival rate close to 100% was noted for every extract tested.

These results suggest that green tobacco extracts do not show a toxicity risk at the concentrations where they exhibit biological activities, supporting their potential safe use in cosmetic applications. However, further examination of these plant extracts using *in vitro* cell-based assays or *in vivo* models would be necessary in the future.

## 3. Materials and Methods

### 3.1. Reagents

Reagents were purchased as follows: sucrose, ethanol, sodium phosphate monobasic, sodium phosphate dibasic, fructose and sodium carbonate (Cicarelli, Santa Fé, Argentina), glucose (Anedra, Nantong, China), choline chloride (Sigma-Aldrich, Beijing, China), urea, ABTS, 4-aminoantipyrine, bromothymol blue, quercetin, Folin–Ciocalteau reagent, and gallic acid (Sigma Aldrich, St. Louis, MO, USA), aluminum chloride (Sigma-Aldrich, Taufkirchen, Germany), agar (Britania, Buenos Aires, Argentina), Coomassie brilliant blue, gelatin type A, collagenase, 4-(dimethylamine) benzaldehyde, potassium tetraborate, sodium hyaluronate, hyaluronidase, N-succinyl-Ala-Ala-Ala-p-nitroanilide, elastase, lipoxygenase, xanthine oxidase (Sigma-Aldrich, St Louis, MO, USA) and linoleic acid (Fluka, Buchs, Switzerland).

### 3.2. Plant Material

Apical fresh leaves of *Nicotiana tabacum* var. Virginia discarded during the budding of the plants (pre-harvest) were collected from crop fields in Perico, Jujuy, Argentina (26°18′ S, 65°37′ W, 1700 m a.s.l.). The samples were dried at 60 °C, up to constant weight in a forced-air stove and grounded in a Helix mill (Metvisa^®^, Mod MP-200-Power 1/2 HP-0.75 Kw, Brusque, Brazil) to obtain leaf powder [19].

### 3.3. NaDESs Preparation

The NaDESs were prepared by mixing the components in the appropriate mole ratios, as follows, fructose:glucose:sucrose:distilled water 1:1:1:11 (FGS) and choline chloride:urea:distilled water 1:2:1.5 (CU). NaDESs were prepared and characterized as described in Leal et al. (2023a) [19]. They were soaked in a water bath at 40 °C, and a stable transparent liquid was obtained after stirring for 20 min [19].

### 3.4. Powder Extraction

Leaf powder was extracted by maceration according to Leal et al. (2023a) [19]. Three green solvents were used: distilled water and the NaDESs fructose:glucose:sucrose (FGS) and choline choride:urea (CU). A plant material: solvent ratio of 1:10 (*w*:*v*) was used. The components were shaken at 100 rpm for 30 min at 25 °C. Then, the extracts were vacuum filtered and stored at −20 °C until use.

### 3.5. Determination of Chemical Composition

#### 3.5.1. Total Polyphenol and Flavonoid Quantification

The different extractive solutions were standardized by the determination of total phenolic compound content according to Singleton et al. (1999) [83]. Different volumes of extracts were mixed with Folin–Ciocalteau reagent, and 15.9% sodium carbonate. The mixture was kept for 20 min at room temperature. The blue color that developed was read at 765 nm in a UV/visible spectrophotometer (Jasco v-630, Thermo Fisher Scientific, Tokyo, Japan). Total flavonoids were measured by a spectrophotometric assay based on aluminum chloride complex formation according to Woisky and Salatino (1998) [84]. The conventional and non-conventional solvent controls were performed in each determination to scan any possible interference. The determinations were performed in triplicate and the results were expressed as µg of gallic acid equivalent (GAE) per mL (µg GAE/mL) and quercetin equivalents (QE) per mL (µg QE/mL), respectively. The extraction yield of each solvent was determined as the amount of chemical components per mL of extract or kg plant material.

#### 3.5.2. UHPLC-MS

The separation and tentative identification of specialized metabolites from *N. tabacum* leaf powder were carried out on a UHPLC-MS system, equipped with UHPLC Ultimate 3000 RS with Metaboscape 4.0 software, and a Bruker maXis ESI-QTOF-MS. The chromatographic equipment consisted of a quaternary pump, an autosampler, a thermostatted column compartment, and a photodiode array detector. The elution was performed by using a binary gradient system with eluent (A) 0.1% formic acid in water, eluent (B) 0.1% formic acid in acetonitrile, and the gradient: isocratic 12% B (0–1 min), 12–99% B (1–15 min), isocratic 99–99% B (15–18 min), 99–12% B (18–18.2 min), 12–12% B (18.2–20 min). The separation was carried out with a Phenomenex Kinetex C18 1.7 μm (2.1 mm × 100 mm) column at a flow rate of 0.4 mL/min. ESI-QTOF-MS experiments in negative ion mode were recorded and the scan range was between 100 and 1200 m/z.

### 3.6. Anti-Inflammatory Activity

In all assays, the concentrations of the samples tested were those in which each of the solvents (NaDESs) themselves did not affect enzyme activity.

#### 3.6.1. Xanthine Oxidase (XO) Inhibition

XO (EC 1.17.3.2) inhibition was determined according to Leal et al. (2021) [85]. Different aliquots of extracts (2–200 μg GAE/mL) with 40 μL of sodium phosphate buffer (200 mM, pH 7.5) and 30 μL of the XO (0.1 U/mL) were pre-incubated at 25 °C for 15 min. The reaction was initiated by adding hypoxanthine (1 mM) and further incubating for 30 min at 25 °C. The enzymatic reaction product was measured at 290 nm with a UV/visible spectrophotometer (Jasco v-630, Thermo Fisher Scientific, Tokyo, Japan). The plotting of the dose–response curves for each extract yielded an expression of concentration (μg GAE/mL) that inhibits 50% of the XO activity; this was named as the inhibitory concentration of 50% of enzyme activity (IC_50_). Allopurinol (2–100 μg/mL) was used as a positive control while distilled water or NaDESs served as the solvent control.

#### 3.6.2. Lipoxygenase (LOX) Inhibition

The inhibition of LOX (EC 1.13.11.12) by *Nicotiana tabacum* leaf extract was determined by Leal et al. (2021) [85]. The process relies on linoleic acid’s enzymatic oxidation to hydroperoxide. The reaction mixture included varying concentrations of the samples, soybean 5-LOX (948 U/mL), sodium borate buffer (200 mM, pH 9.0), and linoleic acid (50 μM), which was incubated at 25 °C for 5 min. The absorbance at 234 nm was measured every 1 min to estimate the amount of hydroperoxide generated with a UV/visible spectrophotometer (Jasco v-630, Thermo Fisher Scientific, Tokyo, Japan). Naproxen was used as the positive control (14 µg/mL), while distilled water or NaDESs served as the solvent control. A regression analysis based on a dose–response curve was used to estimate the concentration required to inhibit 50% of LOX activity (IC_50_).

### 3.7. Activity of Tobacco Leaf Extracts on Skin Aging-Related Enzymes

In all assays, the concentrations of the samples tested were those in which each of the solvents (NaDESs) themselves did not affect enzyme activity.

#### 3.7.1. Elastase Inhibition

Elastase inhibition was determined spectrophotometrically according to Thring et al. (2009) [86], with modifications. Porcine pancreatic elastase (6 µL of 5.6 U/mL) with different concentrations of each extract (89.5 to 295.5 µg GAE/mL) and 0.2 mM Tris-HCl buffer pH 8.0 until final volume of 80 μL were pre-incubated over 15 min at 4 °C. Then, the substrate, 20 µL of N-succinyl-Ala-Ala-Ala-p-nitroanilide (0.36 mg/mL), was added. The reaction mixture was then incubated for 20 min at 25 °C. Epigallocatechin gallate was used as positive control (50 µg/mL), while distilled water or NaDESs served as the solvent control. The absorbance was measured at 410 nm in a microplate (Thermo Scientific Multiskan GO, Vanta, Finland). Regression analysis based on a dose–response curve was used to estimate the concentration of extract or standard required to inhibit 50% of elastase activity (IC_50_).

#### 3.7.2. Hyaluronidase Inhibition

Hyaluronidase activity was spectrophotometrically determined by measuring the amount of N-acetyl glucosamine released from sodium hyaluronate according to Lee et al. (1993) [87], with modifications. Hyaluronidase (5 µL of 0.8 U/mL) was activated by pre-incubation with 20 µL of 0.125 M CaCl_2_ in 0.2 M acetate buffer (pH 4.5) at 37 °C for 10 min. Solvents and designated concentrations of extracts (4.1–53.3 µg GAE/mL) were added to the activated hyaluronidase, and then incubated at 37 °C for 35 min. Sodium hyaluronate (50 µL of 1.33 mg/mL) was subsequently added, and the mixture was incubated at 37 °C for 30 min. The enzymatic reaction was stopped by adding 50 µL of 0.8 M potassium tetraborate, followed by heating in a boiling water bath for 3 min. Then, 1500 µL of 4-(dimethylamine) benzaldehyde 0.01 g/mL (DMAB) was added to the reaction mixture, and incubated at 37 °C for 15 min. The absorbance was measured at 585 nm. Indomethacin was used as positive control (502 µg/mL), while distilled water or NaDESs served as the solvent control. A regression analysis based on a dose–response curve was used to estimate the concentration of extract or standard required to inhibit 50% of hyaluronidase activity (IC_50_).

#### 3.7.3. Collagenase Inhibition

The anti-collagenase activity of *N. tabacum* leaf extracts was determined following the method described by Osathanunkul et al. (2013) [88], with modifications. Briefly, in a 96-well microplate, collagenase enzyme type II from *Clostridium histolyticum* (10 µL of 0.8 U/mL), dissolved in buffer (50 mM Tris-HCl, 10 mM CaCl_2_, and 400 mM NaCl), was pre-incubated at room temperature for 15 min with different concentrations of each extract (103 to 519 µg GAE/mL). Then, 20 µL (13 µg/mL) of gelatin type A was added to the reaction mixture, followed by incubation at 37 °C for 1 h. A volume of 40 µL of Coomassie Brilliant Blue solution (1.25% *w*/*v* in 40% methanol, 10% acetic acid) was added to stain the proteins non-hydrolyzed by the enzyme. The solution was then discarded by pipetting, and the remaining precipitate was dissolved in 100 µL of dimethyl sulfoxide. Absorbance was measured at 600 nm by using a spectrophotometer (Thermo Scientific Multiskan GO, Vanta, Finland). Oleanolic acid was used as positive control (81.9 µg/mL), while distilled water or NADESs served as the solvent control. Regression analysis based on a dose–response curve was used to estimate the concentration of extract or standard required to inhibit 50% of collagenase activity (IC_50_).

### 3.8. Mutagenicity

#### 3.8.1. *Salmonella* Typhimurium Assay

The mutagenic effect of *N. tabacum* extracts was evaluated by using two *Salmonella* Typhimurium strains (TA 98 and TA 100), following the methodology proposed by Maron and Ames (1983) [89]. Briefly, a mixture of 0.1 mL of overnight bacterial culture, different concentrations of extracts (50–500 µg GAE/plate), and 2 mL of top agar was added to minimal agar plates. The plates were then incubated at 37 °C for 48 h, after which the revertant colonies of each plate were counted manually. The mutagenicity relation was calculated using the following formula:

$$Mutagenicity\;ratio=\frac{N{}^{\circ}\;of\;revertant\;colonies\;of\;the\;sample}{N{}^{\circ}\;of\;revertant\;colonies\;of\;the\;negative\;control}$$

The extract was considered mutagenic if the mutagenicity relation was ≥2 [89].

#### 3.8.2. *Caenorhabditis elegans* Toxicity Test

Prior to the trial, a synchronization process of *Caenorhabditis elegans* population was performed. For this purpose, eggs and worms were collected from 3-day-old cultures and treated with alkaline hypochlorite solution (3.16 mL bleach, 5 mL 1N NaOH, 3 mL worm suspension) for 10 min. The mixture was then centrifuged at 1300× *g* for 1 min to obtain an age-synchronized N2 adult population. After multiple washing with M9 buffer, the eggs were transferred onto fresh plates pre-seeded with *Escherichia coli*. Worms were removed from the plates and analyzed after three days of incubation at 20 °C. The assay was performed in a 48-well microplate, with each well containing a final density of 50 worms. To achieve this, worms were suspended in the M9 buffer, and the worm density in the suspension was monitored. The total reaction volume per well was 500 μL, consisting of M9 buffer and varying concentrations of each extract (20, 50, and 100 μg GAE/mL). Survival rate was measured at 20 °C after 24 h and expressed as a percentage of surviving worms [90].

### 3.9. Statistical Analysis

For the statistical analysis of the data, Tukey’s test was applied, with a level of significance *p* < 0.05, by using the statistical package InfoStat V1.1 [91]. A heatmap was devised with Euclidean distance and Ward’s clustering algorithm on the standardized dataset, performed in R software 3.0.2 [92].

## 4. Conclusions

To the best of our knowledge, this is the first study to determine the chemical composition and anti-aging activities of extracts from *Nicotiana tabacum* leaves waste using NaDESs as green solvents. *In vitro* inhibition assays of enzymatic activity against XOD, LOX, hyaluronidase, elastase, and collagenase suggested that green extracts from tobacco leaves exhibited anti-aging properties. In addition, 27 compounds were identified from leaf extracts using the UHPLC-MS approach. Some of these compounds are recognized for their anti-aging properties. Therefore, tobacco leaf waste is a promising natural source of anti-aging ingredients that could be further explored for use in cosmetics and cosmetic–pharmaceutical fields to combat aging and wrinkles. However, further extensive examination of these green extracts *in vitro* cell-based assays or *in vivo* models is required.

## Figures and Tables

**Table 1:** Phenolic compounds and total flavonoid content of *Nicotiana tabacum* var. Virginia leaf waste powder extracts by using conventional solvent (distilled water) and non-conventional solvents (NADESs).

	DW	CU	FGS
Phenolic compounds μg GAE/mL	986.0 ± 19.6 ^a^	2106.0 ± 3.7 ^b^	2402.5 ± 3.7 ^b^
Flavonoids μg QE/mL	18.6 ± 1.8 ^a^	138.4 ± 3.2 ^c^	93.3 ± 7.5 ^b^

GAE: gallic acid equivalent; QE: quercetin equivalent; DW: distilled water; NaDES: FGS, CU; fructose:glucose:sucrose (FGS), choline chloride:urea (CU). Values are reported as mean standard deviation of triplicates. Different letters in the same line indicate significant differences between extracts according to Tukey’s test (*p* < 0.05).

**Table 2:** Chemical compounds tentatively identified in green extracts of apical leaves of *Nicotiana tabacum* using LC-ESI-QTOF-MS/MS. DW-E: aqueous extract; FGS-E: fructose: glucose:sucrose:distilled water extract; CU-E: choline chloride:urea:distilled water extract.

	RT (min)	Theoretical Mass (m/z)	Accuracy (ppm)	Measured Mass (m/z)	Name	[M-H]^−^	Metabolite Type	MS Ions (ppm)	DW-E	FGS-E	CU-E
1	0.37	113.9829	2.3	113.9842	Na formiate (internal standard)	NaC_2_H_2_O_4_	Standard	-			
2	1.83	191.05621	0.598	192.06349	Quinic acid	C_7_H_12_O_6_	Organic acid	127.0399	33,683	54,892	0
3	1.88	133.01434	0.665	134.02163	Malic acid	C_4_H_6_O_5_	Organic acid	115.003, 96.9525	56,926	56,472	47,285
4	1.91	290.08845	1.5	291.09573	N-Fructosyl pyroglutamate	C_11_H_17_NO_8_	Amino acid	111.04517, 93.0354,	8130	18,261	0
5	2.26	191.01999	1.479	192.02727	Citric acid	C_6_H_8_O_7_	Organic acid	179.0217,	2561	10,812	18,591
6	4.01	353.08878	1.928	354.09605	Caffeoyl quinic acid	C_16_H_18_O_9_	Phenolic acid	163.0354, 145.0287	3742	0	0
7	4.75	609.14728	1.505	610.15455	Rutin	C_27_H_30_O_16_	Flavonoid	301.0491,	0	31,265	89,709
8	6.27	175.06133	0.136	176.06862	2-Isopropylmalic acid	C_7_H_12_O_5_	Fatty acid	133.0142, 115.003, 96.9525	49,278	19,727	4222
9	6.58	337.09372	3.045	338.101	Coumaroyl quinic acid	C_16_H_18_O_8_	Phenolic acid	155.0351, 145.0287	7504	4203	0
10	7.12	250.0724	1.007	251.07967	Phenylacetyl aspartic acid	C_12_H_13_NO_5_	Amino acid	177.0404	22,344	13,372	14,175
11	7.12	135.0296	−2.168	136.03688	Threonic acid	C_4_H_8_O_5_	Organic acid	96.1281, 81.6734	1411	9836	0
12	7.28	195.05139	1.86	196.05867	Gluconic acid	C_6_H_12_O_7_	Organic acid	195.0510, 129.0191	34,223	34,047	1875
13	7,72	341.10971	4.914	342.11699	Melibiose	C_12_H_22_O_11_	Glycoside	101.0187, 89.0198,	0	2117	0
14	8.12	132.03085	4.658	133.03812	Aspartate	C_4_H_7_NO_4_	Amino acid	88.0392, 74.0241	2247	33,604	947
15	8.28	117.01954	1.77	118.02682	Succinic acid	C_4_H_6_O_4_	Organic acid	99.0083, 73.0293	2991	59,406	882
16	8.34	147.03117	7.562	148.03845	Citramalic acid	C_5_H_8_O_5_	Organic acid	87.0089	5456	4094	824
17	8.58	179.05343	−22.439	180.06071	Theophylline	C_7_H_8_N_4_O_2_	Alkaloid	124.0325	7662	0	2008
18	8.82	593.15128	0.018	594.15856	Nicotiflorin	C_27_H_30_O_15_	Flavonoid	287.0544, 129.0546	0	4283	9171
19	9.37	187.09756	−0.122	188.10484	Azelaic acid	C_9_H_16_O_4_	Organic acid	95.0481, 57.0356	13,719	7994	15,655
20	9.52	135.04317	−14.716	136.05044	Phenylacetic acid	C_8_H_8_O_2_	Phenolic acid	91.1034	1213	9938	1457
21	10.57	312.12439	1.636	313.13167	Feruloyltyramine	C_18_H_19_NO_4_	Phenolic amide	180–9076, 147.0723	349	0	0
22	10.07	263.12873	−0.852	264.13601	Abscisic acid	C_15_H_20_O_4_	Organic acid	194.0970, 156.0863, 133.0965	3074	3320	3270
23	11.51	201.11302	−1.033	202.1203	Sebacic acid	C_10_H_18_O_4_	Organic acid	183.1033, 139.1138	1236	718	1258
24	12.23	301.03553	0.518	302.04281	Quercetin	C_15_H_10_O_7_	Flavonoid	257.0432, 155.0477,	0	4878	0
25	12.65	285.0408	1.18	286.04807	Kaempferol	C_15_H_10_O_6_	Flavonoid	153.0182, 121.0287	41,899	1462	0
26	11.56	329.06666	0.059	330.07393	Tricin	C_17_H_14_O_7_	Flavonoid	299.0205, 271.0257, 227.0345	701	1356	0
27	13.94	299.2594	−0.85	300.2665	10-Hydroxystearic acid	C_18_H_36_O_3_	Fatty acid	241.0232, 57.0347	1087	1017	3306

**Table 3:** Effect of green extract of *Nicotiana tabacum* apical leaves waste on inflammation-related enzymes.

	XOD	LOX
*N. tabacum* Apical Leaves Waste Extracts	IC_50_ (µg GAE/mL)	IC_50_ (µg GAE/mL)
DW-E	ND	15.20 ± 1.05 ^b^
FGS-E	85.50 ± 2.00 ^c^	17.50 ± 0.83 ^b^
CU-E	63.50 ± 1.10 ^b^	8.0 ± 1.19 ^a^
Naproxen	-	14.0 ± 0.70 ^b^
Allopurinol	50.0 ± 2.0 ^a^	-

DW: Distilled water; FGS: fructose:glucose:sucrose; CU: choline chloride:urea. DW-E: aqueous extract; FGS-E: extract in FGS solvent; CU-E: extract in CU solvent; reference anti-inflammatory agents—allopurinol and naproxen. ND: Not detected. IC_50_: concentration of extract or standard required to inhibit 50% of enzymatic activity. Values are expressed as means ± standard deviation from triplicate measurements. Different letters indicate statistically significant differences among all samples, according to Tukey’s multiple comparison tests (*p* < 0.05).

**Table 4:** Effect of green extracts of *Nicotiana tabacum* apical leaves waste on living model organisms for toxicity analysis.

	*C. elegans* Assay	*S.* Typhimurium Assay	
	% Survival	Mutagenicity Relation	
		TA100	TA98
DW	87.5 ± 2	0.12 ± 0.01	0.12 ± 0.01
DW-E	89.5 ± 5	1.24 ± 0.2	1.02 ± 0.1
FGS	89.4 ± 5	0.16 ± 0.01	0.16 ± 0.01
FGS-E	91.3 ± 3	0.99 ± 0.01	1.42 ± 0.1
CU	81.2 ± 5	0.17 ± 0.01	0.17 ± 0.01
CU-E	95.0 ± 6	1.02 ± 0.1	1.01 ± 0.1

DW: distilled water; FGS: fructose: glucose:sucrose:distilled water; CU: choline chloride:urea. DW-E—aqueous extract; FGS-E—extract in FGS, and CU-E—extract in CU.

## Data Availability

The original contributions presented in this study are included in the article. Further inquiries can be directed to the corresponding author.

## References

[1] Ng M., Freeman M.K., Fleming T.D., Robinson M., Dwyer-Lindgren L., Thomson B., Wollum A., Sanman E., Wulf S., Lopez A.D. (2014). Smoking prevalence and cigarette consumption in 187 countries, 1980–2012. JAMA.

[2] (2025). Datos Argentina. Agroindustria. Tabaco. https://datos.gob.ar/ro/dataset/agroindustria-tabaco---produccion.

[3] Cooperation Centre for Scientific Research Relative to Tobacco (CORESTA). https://www.coresta.org/good-agricultural-practices-gap-guidelines-29207.html.

[4] Diana N.E., Jamil A.H., Yogi Y.A., Nugraheni S.D., Verona L. (2022). Improving the production and quality of Virginia tobacco through topping and suckering: A Review. IOP Conf. Ser. Earth Environ. Sci..

[5] Cosmetic Ingredient Database (CosIng) of the European Commission. https://eur-lex.europa.eu/legal-content/EN/TXT/PDF/?uri=CELEX:32022D0677.

[6] Leffingwell J.C., Davis D.L., Nielsen M.T. (1999). Leaf chemistry. Tobacco: Production, Chemistry and Technology.

[7] Chen Y., Yu Q., Li X., Luo Y., Liu H. (2007). Extraction and HPLC Characterization of Chlorogenic Acid from Tobacco Residuals. Sep. Sci. Technol..

[8] Shifflett J.R., Watson L., McNally D.J., Bezabeh D.Z. (2017). Analysis of the polyphenols of tobacco using pressurized liquid extraction (PLE) and ultra performance liquid chromatography with electrospray ionization–tandem mass spectrometric detection (UPLC-ESI-MS/MS). CTNR.

[9] Sifola M.I., Carrino L., Cozzolino E., del Piano L., Graziani G., Ritieni A. (2021). Potential of Pre-Harvest Wastes of Tobacco (*Nicotiana tabacum* L.) Crops, Grown for Smoke Products, as Source of Bioactive Compounds (Phenols and Flavonoids). Sustainability.

[10] Zou X., Bk A., Rauf A., Saeed M., Al-Awthan Y.S., Al-Duais M., Bahattab O., Hamayoon Khan M., Suleria H.A. (2021). Screening of polyphenols in tobacco (*Nicotiana tabacum*) and determination of their antioxidant activity in different tobacco varieties. ACS Omega.

[11] Banožic M., Babi´c J., Joki´c S. (2020). Recent advances in extraction of bio-active compounds from tobacco industrial waste-a review. Ind. Crops Prod..

[12] Severson R.F., Arrendale R.F., Chortyk O.T., Johnson A.W., Jackson D.M., Gwynn G.R., Chaplin J.F., Stephenson M.G. (1984). Quantitation of the major cuticular components from green leaf of different tobacco types. J. Agric. Food Chem..

[13] Sisson V.A., Severson R.F. (1990). Alkaloid composition of the Nicotiana species. Beitr. Tab. Int..

[14] Chemat F., Abert-Vian M., Fabiano-Tixier A.S., Strube J., Uhlen-brock L., Gunjevic V., Cravotto G. (2019). Green extraction of natural products. Origins, current status, and future challenges. TrAC Trends Anal. Chem..

[15] Villa C., Caviglia D., Robustelli della Cuna F.S., Zuccari G., Russo E. (2024). NaDES Application in Cosmetic and Pharmaceutical Fields: An Overview. Gels.

[16] Benoit C., Virginie C., Boris V. (2021). Chapter Twelve -The Use of NADES to Support Innovation in the Cosmetic Industry. Adv. Bot. Res..

[17] Pena-Pereira F., Kloskowski A., Namiesnik J. (2015). Perspectives on the Replacement of Harmful Organic Solvents in Analytical Methodologies: A Framework toward the Implementation of a Generation of Eco-Friendly Alternatives. Green Chem..

[18] Roccha D., Freitas D.S., Magalhães J., Fernandes M., Silva S., Noro J., Ribeiro A., Cavaco-Paulo A., Martins M., Silva C. (2023). NADES-Based Cork Extractives as Green Ingredients for Cosmetics and Textiles. Processes.

[19] Leal M., Moreno M.A., Albornoz P.L., Mercado M.I., Zampini I.C., Isla M.I. (2023). *Nicotiana tabacum* leaf waste: Morphological characterization and chemical-functional analysis of extracts obtained from powder leaves by Using Green Solvents. Molecules.

[20] Leal M., Moreno M.A., Albornoz P.L., Mercado M.I., Zampini I.C., Isla M.I. (2023). Morphological Characterization of *Nicotiana tabacum* Inflorescences and Chemical-Functional Analysis of Extracts Obtained from Its Powder by Using Green Solvents (NaDESs). Plants.

[21] Shin S.H., Lee Y.H., Rho N.K., Park K.Y. (2023). Skin aging from mechanisms to interventions: Focusing on dermal aging. Front. Physiol..

[22] Banglao W., Thongmee A., Sukplang P., Wanakhachornkrai O. (2020). Determination of antioxidant, anti-aging and cytotoxicity activity of the essential oils from *Cinnamomum zeylanicum*. J. Microbiol. Biotechnol. Food Sci..

[23] Abd Razak D.L., Jamaluddin A., Abd Rashid N.Y., Sani N.A., Abdul Manan M. (2020). Assessment of cosmeceutical potentials of selected mushroom fruitbody extracts through evaluation of antioxidant, anti-hyaluronidase and anti-tyrosinase activity. J.

[24] Nworu C.S., Akah P.A. (2015). Anti-inflammatory medicinal plants and the molecular mechanisms underlaying their activities. Afr. J. Tradit. Complement. Altern. Med..

[25] Perfetti T.A., Rodgman A. (2011). The complexity of tobacco and tobacco smoke. Beitr. Tabakforsch. Int..

[26] Ncube E.N., Mhlongo M.I., Piater L.A., Steenkamp P.A., Dubery I.A., Madala N.E. (2014). Analyses of chlorogenic acids and related cinnamic acid derivatives from *Nicotiana tabacum* tissues with the aid of UPLC-QTOF-MS/MS based on the in-source collision-induced dissociation method. Chem. Cent. J..

[27] Matsumoto T., Mikami Y., Tomita H. (1976). Isolation of 2 Isopropyl-4,4-dimethyl-5-vinylidene-2-cyclopenten-1-one and 2-Isopropyl-3-isopentyl-maleic Anhydride from the Pyrolytic Products of 2-Isopropylmalic acid. Agric. Biol. Chem..

[28] Zhang X., Peng J., Zhang L., Long M., Zuo J. (2013). Optimization of microwave drying biomass material of stem granules from waste tobacco using response surface methodology. Dry. Technol..

[29] Zhang J., Zhang Y., Du Y., Chen S., Tang H. (2011). Dynamic metabonomic responses of tobacco (*Nicotiana tabacum*) plants to salt stress. J. Proteome Res..

[30] Jing Y., Zhang B., Yuan X., Gao Y., Lu P., Wang W., Xu M. (2016). Determination of free amino acids in burley tobacco by high performance liquid chromatography. Saudi J. Biol. Sci..

[31] Wightman F., Lighty D.L. (1982). Identification of phenylacetic acid as a natural auxin in the shoots of higher plants. Physiol. Plant..

[32] Negrel J., Martin C. (1984). The biosynthesis of feruloyltyramine in *Nicotiana tabacum*. Phytochemistry.

[33] Whenham R.J., Fraser R.S.S., Brown L.P., Payne J.A. (1986). Tobacco-mosaic-virus-induced increase in abscisic-acid concentration in tobacco leaves: Intracellular location in light and dark-green areas, and relationship to symptom development. Planta.

[34] Nagy Z.Á., Kátay G., Gullner G., Király L., Ádám A.L. (2017). Azelaic acid accumulates in phloem exudates of TMV-infected tobacco leaves, but its application does not induce local or systemic resistance against selected viral and bacterial pathogens. Acta Physiol. Plant..

[35] Yang C., Xie S.N., Ni L., Du Y.M., Liu S., Li M.Y., Xu K. (2021). Chemical constituents from *Nicotiana tabacum* L. and their antifungal activity. Nat. Prod. Commun..

[36] de Macêdo I.S.V., Cunha K.G., Alves A.T.V., Martins R.M., da Silva Simões M.O. (2007). Atividade antioxidante da rutina: Uma revisão. BioFarm.

[37] Girsang E., Lister I.N.E., Ginting C.N., Sholihah I.A., Raif M.A., Kunardi S., Million H., Widowati W. (2020). Antioxidant and antiaging activity of rutin and caffeic acid. Pharmaciana.

[38] Dehaghani Z.A., Asghari G., Dinani M.S. (2007). Isolation and identification of nicotiflorin and narcissin from the aerial parts of *Peucedanum aucheri* Boiss. J. Agric. Sci. Technol. A.

[39] Liana L., Rizal R., Widowati W., Fioni F., Akbar K., Fachrial E., Lister I.N.E. (2019). Antioxidant and anti-hyaluronidase activities of dragon fruit peel extract and kaempferol-3-o-rutinoside. JKB.

[40] Singh S.K., Chaubey S., Bansal A., Kaur G., Malik D.S. (2021). Cosmeceutical aptitudes of azelaic acid. Curr. Drug Res. Rev..

[41] Sieber M.A., Hegel J.K.E. (2013). Azelaic acid: Properties and mode of action. Ski. Pharmacol. Physiol..

[42] Fitton A., Goa K.L. (1991). Azelaic acid: A review of its pharmacological properties and therapeutic efficacy in acne and hyperpigmentary skin disorders. Drugs.

[43] Quaresma S., André V., Antunes A.M., Vilela S.M., Amariei G., Arenas-Vivo A., Rosal R., Horcajada P., Duarte M.T. (2019). Novel antibacterial azelaic acid BioMOFs. Cryst. Growth Des..

[44] Ercan L., Doğru M. (2022). Antioxidant and antimicrobial capacity of quinic acid. Bitlisfen.

[45] Zeng K., Thompson K.E., Yates C.R., Miller D.D. (2009). Synthesis and biological evaluation of quinic acid derivatives as anti-inflammatory agents. Bioorg. Med. Chem. Lett..

[46] Choi J.Y., Lee J.W., Jang H., Kim J.G., Lee M.K., Hong J.T., Lee M.S., Hwang B.Y. (2021). Quinic acid esters from *Erycibe obtusifolia* with antioxidant and tyrosinase inhibitory activities. Nat. Prod. Res..

[47] Kazemi M., Hadavi E., Hekmati J. (2012). Effect of salicylic acid, malic acid, citric acid and sucrose on antioxidant activity, membrane stability and ACC-Oxidase activity in relation to vase life of carnation cut flowers. J. Agric. Technol..

[48] Singh S.K., Kaldate R., Bisht A. (2022). Citric acid, antioxidant effects in health. Antioxidants Effects in Health. The Bright and the Dark Side.

[49] Ramachandran S., Fontanille P., Pandey A., Larroche C. (2006). Gluconic acid: Properties, applications and microbial production. FTB.

[50] Periferakis A., Periferakis K., Badarau I.A., Petran E.M., Popa D.C., Caruntu A., Costache R.S., Scheau C., Caruntu C., Costache D.O. (2022). Kaempferol: Antimicrobial properties, sources, clinical, and traditional applications. Int. J. Mol. Sci..

[51] Rho H.S., Ghimeray A.K., Yoo D.S., Ahn S.M., Kwon S.S., Lee K.H., Cho D.H., Cho J.Y. (2011). Kaempferol and kaempferol rhamnosides with depigmenting and anti-inflammatory properties. Molecules.

[52] Aghababaei F., Hadidi M. (2023). Recent advances in potential health benefits of quercetin. Pharmaceuticals.

[53] Azeem M., Hanif M., Mahmood K., Ameer N., Chughtai F.R.S., Abid U. (2023). An insight into anticancer, antioxidant, antimicrobial, antidiabetic and anti-inflammatory effects of quercetin: A review. Polym. Bull..

[54] Moon-Hee C., Shin H.J. (2016). Anti-Melanogenesis Effect of Quercetin. Cosmetics.

[55] Mu L.L., Kou J.P., Zhu D.N., Yu B.Y. (2008). Antioxidant activities of the chemical constituents isolated from the leaves of Ginkgo biloba. CJNM.

[56] Cai H., Sale S., Schmid R., Britton R.G., Brown K., Steward W.P., Gescher A.J. (2009). Flavones as colorectal cancer chemopreventive agents—Phenol-O-methylation enhances efficacy. Cancer Prev. Res..

[57] Quan N.V., Thien D.D., Khanh T.D., Tran H.D., Xuan T.D. (2019). Momilactones A, B, and tricin in rice grain and by-products are potential skin aging inhibitors. Foods.

[58] Liu W., Li J., Zhang X., Zu Y., Yang Y., Liu W., Xu Z., Gao H., Sun X., Jiang X. (2020). Current advances in naturally occurring caffeoylquinic acids: Structure, bioactivity, and synthesis. J. Agric. Food Chem..

[59] Alcázar Magaña A., Kamimura N., Soumyanath A., Stevens J.F., Maier C.S. (2021). Caffeoylquinic acids: Chemistry, biosynthesis, occurrence, analytical challenges, and bioactivity. Plant J..

[60] Zhu Y.J., Zhou H.T., Hu Y.H., Tang J.Y., Su M.X., Guo Y.J., Chen Q.L., Liu B. (2011). Antityrosinase and antimicrobial activities of 2-phenylethanol, 2-phenylacetaldehyde and 2-phenylacetic acid. Food Chem..

[61] Rawlings A.V., Wandeler E., Bendik I., Fuchs P., Monneuse J.M., Imfeld D., Schütz R. (2021). Effect of regioisomers of hydroxystearic acids as peroxisomal proliferator-activated receptor agonists to boost the anti-ageing potential of retinoids. Int. J. Cosmet. Sci..

[62] Mhiri R., Koubaa I., Chawech R., Auberon F., Allouche N., Michel T. (2020). New isoflavones with antioxidant activity isolated from *Cornulaca monacantha*. Chem. Biodivers..

[63] Jiang Y., Yu L., Wang M.H. (2015). N-trans-feruloyltyramine inhibits LPS-induced NO and PGE2 production in RAW 264.7 macrophages: Involvement of AP-1 and MAP kinase signalling pathways. Chem. Biol. Interact..

[64] Ricciutelli M., Bartolucci G., Campana R., Salucci S., Benedetti S., Caprioli G., Maggi F., Sagratini G., Vittori S., Lucarini S. (2020). Quantification of 2-and 3-isopropylmalic acids in forty Italian wines by UHPLC-MS/MS triple quadrupole and evaluation of their antimicrobial, antioxidant activities and biocompatibility. Food Chem..

[65] Union European (2009). Regulation (EC) No 1223/2009 of the European Parliament and of the Council. Off. J. Eur. Union.

[66] Agrawal R., Hu A., Bollag W.B. (2023). The Skin and Inflamm-Aging. Biology.

[67] Mangerich A., Dedon P.C., Fox J.G., Tannenbaum S.R. (2013). Chemistry meets biology in colitis-associated carcinogenesis. Free Radic. Res..

[68] Isla M.I., Ezquer M.E., Leal M., Moreno M.A., Zampini I.C. (2021). Flower beverages of native medicinal plants from Argentina (*Acacia caven*, *Geoffroea decorticans* and *Larrea divaricata*) as antioxidant and anti-inflammatory. J. Ethnopharmacol..

[69] Naveed M., Hejazi V., Abbas M., Kamboh A.A., Khan G.J., Shumzaid M., Ahmad F., Babazadeh D., Xia F., Modarresi-Ghazani F. (2018). Chlorogenic acid (CGA): A pharmacological review and call for further research. Biomed. Pharmacother..

[70] Asmi K.S., Lakshmi T., Balusamy S.R., Parameswari R. (2017). Therapeutic aspects of taxifolin–An update. J. Adv. Pharm. Educ. Res..

[71] Guardia T., Rotelli A.E., Juarez A.O., Pelzer L.E. (2001). Anti-inflammatory properties of plant flavonoids. Effects of rutin, quercetin and hesperidin on adjuvant arthritis in rat. Il Fármaco.

[72] Rajendran P., Rengarajan T., Nandakumar N., Palaniswami R., Nishigaki Y., Nishigaki I. (2024). Kaempferol, a potential cytostatic and cure for inflammatory disorders. Eur. J. Med. Chem..

[73] Naylor E.C., Watson R.E., Sherratt M.J. (2011). Molecular aspects of skin ageing. Maturitas.

[74] Papakonstantinou E., Roth M., Karakiulakis G. (2012). Hyaluronic acid: A key molecule in skin aging. Dermato-endocrinology.

[75] Prommaban A., Kheawfu K., Chittasupho C., Sirilun S., Hemsuwimon K., Chaiyana W. (2022). Phytochemical, antioxidant, antihyaluronidase, antityrosinase, and antimicrobial properties of *Nicotiana tabacum* L. leaf extracts. eCAM.

[76] Xu Y., Chen G., Guo M. (2022). Potential Anti-aging Components from *Moringa oleifera* Leaves Explored by Affinity Ultrafiltration with Multiple Drug Targets. Front. Nutr..

[77] Kinari S., Girsang E., Nasution A.N., Lister I.N.E. (2019). Antioxidant and anti-Collagenase effectivity of red dragon fruit peel and Kaempferol 3-0-Rutinoside. ASRJETS.

[78] Kuzniewski R., Załuski D., Olech M., Banaszczak P., Nowak R. (2018). LC-ESI-MS/MS profiling of phenolics in the leaves of *Eleutherococcus senticosus* cultivated in the West Europe and anti-hyaluronidase and anti-acetylcholinestarase activities. Nat. Prod. Res..

[79] Xinghua L., Yingying H., Shuai W. (2023). Anti-aging Effect of Rutin in *Caenorhabditis elegans* and D-Gal-Induced Aging Mouse Model. Dokl. Biochem. Biophys..

[80] Matsunami K. (2018). Frailty and *Caenorhabditis elegans* as a benchtop animal model for screening drugs including natural herbs. Front. Nutr..

[81] Choi J., Ahn A., Kim S., Won C.W. (2015). Global prevalence of physical frailty by fried’s criteria in community-dwelling elderly with national population-based surveys. J. Am. Med. Dir. Assoc..

[82] David D.C., Ollikainen N., Trinidad J.C., Cary M.P., Burlingame A.L., Kenyon C. (2010). Widespread protein aggregation as an inherent part of aging in *C. elegans*. PLoS Biol..

[83] Singleton V.L., Rossi J.A. (1965). Colorimetry of total phenolics with phosphomolybdic-phosphotungstic acid reagents. Am. J. Enol. Vitic..

[84] Woisky R.G., Salatino A. (1998). Analysis of propolis: Some parameters and procedures for chemical quality control. J. Apic. Res..

[85] Leal M., Zampini C., Mercado M.I., Moreno M.A., Simirgiotis M., Borquez J., Ponessa G., Isla M.I. (2021). *Flourensia fiebrigii* S.F. blake: A medicinal plant from the Argentinean highlands with potential use as anti-rheumatic and anti-inflammatory. J. Ethnopharmacol..

[86] Thring T.S., Hili P., Naughton D.P. (2009). Anti-collagenase, anti-elastase and anti-oxidant activities of extracts from 21 plants. BMC Complement. Altern. Med..

[87] Lee J., Lee S.H., Min K.R., Lee K.S., Ro J.S., Ryu J.C., Kim Y. (1993). Inhibitory effects of hydrolyzable tannins on Ca^2+^-activated hyaluronidase. Planta Med..

[88] Osathanunkul M., Buddhachat K., Chomdej S. (2023). A modified colorimetric method of gelatinolytic assay using bacterial collagenase type II as a model. Anal. Biochem..

[89] Maron D.M., Ames B.N. (1983). Revised methods for the Salmonella mutagenicity test. Mutat. Res. Genet. Toxicol. Environ. Mutagen..

[90] Orqueda M.E., Zampini I.C., Torres S., Isla M.I. (2023). Functional Characterization and Toxicity of Pectin from Red Chilto Fruit Waste (Peels). Plants.

[91] Di Rienzo J.A., Casanoves F., Balzarini M.G., Gonzalez L., Tablada M., Robledo C.W. (2015). InfoStat.

[92] RStudio Team (2020). RStudio: Integrated Development for R.

